# Guessing can benefit memory for related word pairs even when feedback is delayed

**DOI:** 10.3758/s13421-022-01385-0

**Published:** 2023-01-12

**Authors:** Katarzyna Zawadzka, Oliwia Zaborowska, Ewa Butowska, Krzysztof Piątkowski, Maciej Hanczakowski

**Affiliations:** 1 Adam Mickiewicz University, ul. Szamarzewskiego 89AB, 60-568 Poznań, Poland; 2 SWPS University, ul. Chodakowska 19/31, 03-815 Warsaw, Poland

**Keywords:** Testing, Feedback, Retrieval, Judgments of learning

## Abstract

Trying to guess what the correct answer to a question might be can facilitate future learning of this answer when presented in the form of corrective feedback. One issue that determines the effectiveness of guessing as a learning strategy is the timing of the presentation of feedback: it can be presented either immediately after the guess, or after a delay. Whereas the timing of feedback is of little importance for complex materials such as trivia questions, previous research suggests that for simpler materials such as related word pairs guessing seems to benefit learning only when feedback is immediate. In order to test whether this always has to be the case, we conducted two experiments in which we increased the richness of study materials by superimposing the to-be-learned word pairs over unrelated context pictures. We then manipulated the match between contexts at study and at test (Experiment 1) and at the time of feedback delivery (Experiment 2). Contrary to previous studies showing no benefits of guessing with delayed feedback, our results show that learning related word pairs can benefit from guessing even when feedback is delayed. These benefits of guessing occur if participants are reminded via reinstated contexts of the guessing stage at the time of feedback delivery. Our results help constrain theories of guessing benefits and extend theories of reminding.

Testing is a ubiquitous feature of learning. Classroom tests have long been used as means of assessing students’ knowledge, but there is now also a growing realization that, in addition to assessment, testing one’s memory can also be used to gain knowledge (Roediger & Karpicke, 2006; Rowland, 2014). It is thus important to understand how and under what circumstances attempting to retrieve answers from memory can be used to facilitate learning.

A recent line of studies has demonstrated that retrieval attempts can be beneficial even when correct answers are unlikely to be produced, as long as corrective feedback is presented after the provision of initial responses. In a typical procedure, participants are either presented with some materials (e.g., facts, weakly related pairs of words) to read and learn, or are first presented with parts of these materials (e.g., questions, first words from the pairs) and asked to guess what the remaining part (i.e., the answer to the question, or the second word from the related pair) might be before being presented with it for study. As shown numerous times since the study by Kornell et al. (2009) which introduced this guessing paradigm in its current formulation, guessing followed by corrective feedback can improve memory for to-be-learned materials compared with merely reading them even when participants’ initial guesses are incorrect (e.g., Bridger & Mecklinger, 2014; Grimaldi & Karpicke, 2012; Huelser & Metcalfe, 2012; Knight et al., 2012; Kornell, 2014; Vaughn & Rawson, 2012; but see Seabrooke et al., 2019, for limitations).

If guessing improves memory for corrective feedback, then the timing of this feedback becomes a question of high importance, both theoretical and practical. Given that any effective learning strategy should ultimately be suited for use in actual educational settings, it is important to consider when feedback can be viably delivered. If a question is posed informally in the classroom, for example as part of a discussion between the instructor and the group, and students are asked to guess what the answer to this question might be, then feedback can be delivered by the instructor immediately after the guessing attempt. But when questions are administered in the form of a formal educational test, then feedback is often delivered after a delay ranging from minutes to even weeks. In this context, it has to be noted that the majority of studies showing the benefits of guessing have done so when feedback was immediate rather than delayed. In fact, out of four studies which compared the two feedback timings, three (Grimaldi & Karpicke, 2012; Hays et al., 2013; Vaughn & Rawson, 2012) have shown no benefits of delayed feedback, and sometimes even costs to learning, compared with a condition that did not involve any guessing. At the same time, when feedback was immediate, they documented the typical benefits of guessing over reading. All three of these studies used related word pairs (e.g., *pond–frog*) as study materials. The focus of the fourth study (Kornell, 2014) was on the influence of the type of to-be-learned materials on the effectiveness of learning the corrective feedback in the guessing paradigm. In Experiment 1, Kornell (2014) replicated the lack of guessing benefits with delayed feedback when related word pairs were used. In subsequent experiments, study materials were changed to trivia questions. With those materials, the benefits of guessing emerged both when feedback was immediate and when it was delayed, and were present even when there was a 24-hour delay between guessing and the provision of feedback.

One reason suggested by Kornell (2014) as a potential explanation for why the benefits of guessing with delayed feedback can be found for trivia questions but not for word pairs has to do with the fact that the representation of the cues provided at the time of guessing are subjected to semantic elaboration. Here we use the umbrella term *semantic elaboration* to refer to the processes that build on access to information semantically related to cues at the time when guesses are formulated. This idea is closely related to Carpenter’s (2009, 2011) elaborative retrieval hypothesis, according to which being presented with a retrieval cue and attempting to retrieve the target activates information semantically related to the cue, including possible candidate answers; this activation is thought to underlie the benefits of testing over restudy in terms of memory performance on the final test. When people guess at an answer, semantic activation may result in activation of the correct response, which then allows for associating this response with already available knowledge structures (e.g., Bridger & Mecklinger, 2014; Grimaldi & Karpicke, 2012). It may also facilitate generation of other candidate answers, which can later be used as semantic mediators, such as associates of both the cue and the target (e.g., cue: *mother*, mediator: *father*, target: *child*; Carpenter, 2011; Vaughn & Rawson, 2012) or episodic mediators such as guesses based on processing of the cue (cues: *tree–palm*; guess: *coconut*; target: *hand*; Metcalfe & Huelser, 2020). Whatever the type of mediators, they facilitate linking the questions and their respective correct answers, aiding subsequent retrieval at test.

Importantly, Kornell (2014) hypothesized that this semantic activation arising from guessing is something that can at least under some circumstances be reinstated after a delay. Comparing trivia questions to single words serving as cues, he argued that processing questions is more likely to result in creation of distinctive long-term memory representations. When re-presented later in the course of the experiment, these distinctive representations of trivia questions increase the chances that participants would think back to what they thought of at the time of the initial presentation of the same question, potentially reintroducing the same pattern of semantic activation, and thus conferring benefits on memory performance. Single-word cues, on the other hand, were thought by Kornell to be less likely to create at the time of their initial elaboration memory traces that would be distinctive enough to recreate the original activation when re-presented after a delay.

If this hypothesis proposed by Kornell (2014) is correct, it would suggest that there is no fundamental impediment to observing the benefits of guessing for related pairs of words even when feedback is delayed. The precondition for these benefits, however, would be that the same activation, which normally accrues at the time of guessing, would be reinstated from long-term memory when necessary. If the reinstated activation from the time of guessing were to arise at the time of the final test, it could then allow participants to retrieve the mediators generated at the guessing stage, helping cue the correct answer. If it were to arise at the time of feedback delivery, it could facilitate encoding of corrective feedback by associating feedback with the activated knowledge structures.

The aim of the present study is thus to examine whether it is possible to create conditions under which the benefits of guessing emerge when related pairs of words are used as study materials and feedback is delayed. The approach adopted here is to reinstate the processes engaged at the time of guessing at later phases of the guessing paradigm via a well-known manipulation of context reinstatement. Context reinstatement refers to reusing the same context that accompanied the initial presentation of a studied item at a later stage of a memory task, for example at restudy (e.g., Saenz & Smith, 2018; Smith & Handy, 2014; Zawadzka et al., 2018) or at test (e.g., Hanczakowski et al., 2014; Murnane et al., 1999; Reder et al., 2013). Reinstated contexts tend to improve retrieval of the original episode compared with contexts that are novel—that is, have no pre-existing association with any of the studied items. This augmented retrieval may come as explicit recollection of the original episode as reflected in recognition (e.g., Hockley, 2008; Macken, 2002) or recall (e.g., Smith & Manzano, 2010) performance, but also as more automatic activation that influences performance in indirect memory tests (Smith et al., 2018). Whether by the intentional or the automatic route, it is thus viable that context reinstatement would bring back similar semantic activation or processing to that which occurred when the original context was first presented. If so, then bringing back the semantic components from the stage of guessing via context reinstatement might allow for the benefits of guessing to emerge with related word pairs even when feedback is delayed.

We reasoned that there are two stages of the memory process at which the effectiveness of the guessing strategy with delayed feedback can be undermined. First, it might be that for related word pairs it is more difficult to capitalize on the guessing process at the time of test. Here, it is important to note that while only a single memory representation of studying a pair of words is created via reading or via guessing with immediate feedback, with delayed feedback two memory representations can be potentially established, with the first one containing the details of the guessing event and the second one containing the details of feedback processing. If participants access at test only the representation containing the details of feedback processing, this would be no more effective than accessing a single representation generated via reading. If they access only the representation containing the details of the guessing event, it would not help them retrieve the correct answer as it was never presented at that stage of the experiment. Conversely, if both representation are accessed at the same time, participants should be able to benefit from the access to mediators generated at the time of guessing, which would then help cue the correct answer presented at the time of feedback delivery. Thus, the argument here would be that being able to access both memory representations at test is a necessary precondition for observing the guessing benefits when feedback is delayed. This hypothesis was assessed in Experiment 1 by manipulating the match in terms of contextual cues present at retrieval. Thus, these cues either matched or mismatched the context present at study, both at the time of guessing and at the time of feedback delivery.

Second, the lack of guessing benefits for related pairs when feedback is delayed might stem from impaired feedback processing. Here, guessing does not improve memory because by the time the feedback is presented, the semantic activation resulting from the guessing processes has already faded. It could thus be predicted that if the processes operating at the guessing stage could be reinstated when feedback is provided, this would result in enhanced memory compared with the read condition. Experiment 2 directly tested this hypothesis by manipulating the match in terms of contextual cues at feedback processing, which either matched or mismatched the context present during guessing.

## Experiment 1

In Experiment 1, participants studied pairs of weakly related words in three different conditions. In the *read* condition, participants were presented with full pairs for study and made no guessing attempt. In the *immediate-feedback* condition, participants were first presented with cues only and asked to guess their respective targets, and then were immediately presented with the full pair for study. In the *delayed-feedback* condition, the feedback was presented several minutes after the guessing attempt. In addition to manipulating guessing, we also introduced context photographs at study—all items were presented superimposed over unique photographs of buildings, landscapes, animals, and so forth—and we manipulated contexts between study and test. The individual contexts with which particular study pairs were presented were always held constant during the whole study phase. Thus, for both the immediate-feedback and delayed-feedback conditions, first cues and then cues with their respective targets were presented in the same context. The context manipulation was introduced at test: Half of all items in each condition were tested with the cues accompanied by the same contexts which were present at study—the *reinstated-context* condition—and half were tested with the cues accompanied by novel contexts, not seen before—the *novel-context* condition. The aim of this experiment was to assess whether reinstating contexts at test would specifically impact retrieval in the delayed-feedback condition, facilitating access to potential mediators created during study, and thus improving performance over and above the performance levels in the read condition.

In addition, we looked at metacognitive monitoring of encoding in the form of judgments of learning (JOLs). Recent investigations into the benefits of guessing have revealed that even though this strategy is effective in augmenting memory—at least when immediate feedback is provided—participants lack metacognitive insight into these benefits (Huelser & Metcalfe, 2012; Potts & Shanks, 2014; Yang et al., 2017; Zawadzka & Hanczakowski, 2019). Specifically, participants consistently predict that the likelihood of remembering pairs for which guesses are formulated is lower than the likelihood of remembering pairs which are presented outright, even though later memory performance suggests the opposite. This pattern creates problems if one is interested in promoting guessing as a learning strategy because if people do not see this strategy as effective, they may be reluctant to adopt it. At present it is unknown how people appraise guessing under delayed-feedback conditions. For this reason, we collected immediate JOLs after the presentation of each full pair.^1^

## Method

### Participants

Sixty students (age range: 18–56 years, *M* = 20.5) of the University of Sheffield, UK, participated in this experiment in exchange for course credit or monetary compensation.

#### Materials and design

A total of 246 words were chosen from the University of South Florida Free Association Norms (Nelson et al., 1998). One hundred and twenty-three of these words were designated to serve as cues. For each of those words, a weakly related (on average .05, which means that the probability of spontaneously producing this word in response to a given cue was 5%) word was chosen as a target. Three of the pairs were reserved for the practice task. The remaining 120 word pairs were further subdivided into two lists of 60 pairs, each of which was assigned to one study-test block.

**Fig. 1:**
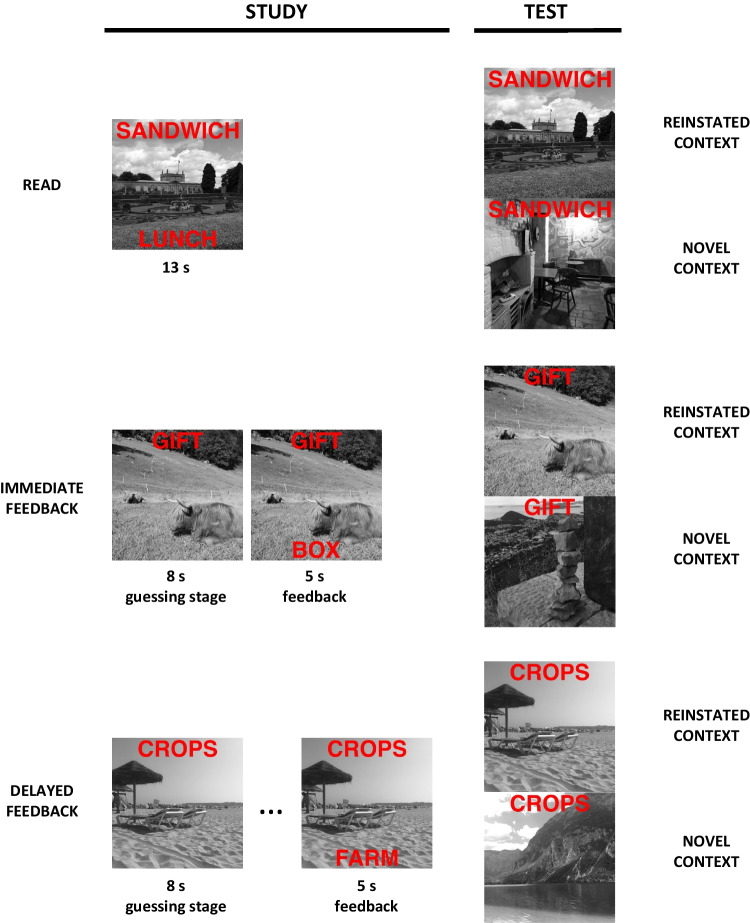
A schematic design of a single experimental block in Experiment 1. Guessing conditions are indicated on the left-hand side, and context conditions on the right-hand side

A schematic design of the experiment is presented in Fig. 1. Within each list, each word pair was assigned to one of three learning conditions, which was counterbalanced across participants. In the read condition, the full pair was presented to participants for 13 seconds for study. In both feedback conditions, participants were first presented only with the cue and were asked to guess a word related to that cue within eight seconds. Then, in the immediate-feedback condition, the designated target word was presented together with the cue for 5 seconds. In the delayed-feedback condition, this feedback was preceded by other study and guessing trials. In order to achieve this, unbeknownst to participants the study phase was split into two parts. In the first part, all 20 cues from the delayed-feedback condition were presented for guessing, accompanied by five trials each from the read and immediate-feedback condition, all in random order. In the second part, feedback was delivered for all pairs from the delayed-feedback condition, and participants were also presented with the remaining pairs from the read and immediate-feedback conditions, with the presentation order again being randomized. Thus, the average delay between the presentation of a pair in the delayed-feedback condition and the presentation of feedback for this pair was 40 trials.^2^

A set of 183 black-and-white pictures was used as contexts for the study materials. Three pictures were chosen for the practice task. The remaining set of 180 pictures was then divided into two halves, and each of the halves was assigned to one study–test block. Within each block, the pictures were further subdivided into one set of 60 and one of 30. One picture from each 60-picture set was then assigned at random to one word pair. The pictures from this set were used for the study phase. In the two feedback conditions, they were presented to participants at the time of guessing with cues superimposed over the top portion of the picture. In addition to that, in all three conditions full word pairs were presented superimposed over those pictures for study, with the cue at the top and the target at the bottom. All words were written in red capital letters. Both sets of pictures were then used for test. For half of the word pairs, cues at test were presented at the top of the same picture that was presented with the full pair at study. These constituted the reinstated-context condition. For the other half, cues were presented superimposed over new pictures, not presented before in any phase of the experiment—the novel-context condition.

#### Procedure

Participants were first informed that they would see word pairs presented over unrelated black-and-white photographs, without any explicit instructions regarding these photographs. They were told that at study, they would encounter three types of trials. For the first trial type, they would be presented with full pairs of words and their task would be to memorize these pairs within 13 seconds. For the second trial type, they would see the first word only and they would have to guess what the second word might be within 8 seconds. After that time, they would see the full pair—the first word together with its correct answer—and they would have five seconds to learn that pair. For the third trial type, they would also have to guess the second word within 8 seconds, but they would only be shown the full pair at a later stage for five seconds. They were also told that after the presentation of each full pair they would be asked for their confidence—on a scale from 1 to 5—in recalling the second word at test in approximately 10 minutes when presented with the first one—that is, make a JOL. They then completed a short practice task for all types of trials as well as JOLs under the supervision of the experimenter. After the practice phase, participants were informed about the subsequent test, which would require them to type in the second words from each pair (and not their guesses) in response to the first words. They were also told that the study and test procedure would then be repeated with new materials. In the first study phase, a list of 60 words was presented, consisting of 20 words assigned to the read condition, 20 to the immediate-feedback condition, and 20 to the delayed-feedback condition. After the study phase, participants were once again provided with instructions regarding the upcoming test. In those instructions, there was no mention of the background pictures. The test was self-paced. Cue words were presented in random order one at a time to participants, and the task was to type in the corresponding target or press Enter if no response was provided to advance to the next cue. Thirty cues were assigned to the reinstated-context condition and 30 to the novel-context conditions—10 each from the read, immediate-feedback, and delayed-feedback conditions. The test was followed by the second study–test block, which was identical to the first one, barring the change of all materials.

## Results and discussion

Descriptive statistics for cued-recall performance are presented in Table 1. Additionally, box and violin plots can be found in the Appendix Figs. 3 and 4. A 2 (context: reinstated, novel) × 3 (learning condition: delayed-feedback, immediate-feedback, read) repeated-measures analysis of variance (ANOVA) performed on accuracy data revealed a significant main effect of context, *F*(1, 59) = 6.18, *p* = .016, η_p_^2^ = .095, with performance being higher when items were accompanied at test by the same contexts with which they were paired at study—that is, in the reinstated-context condition (*M* = .66, *SD* = .18)—than when the contexts used at test were novel (*M* = .64, *SD* = .18). The main effect of the learning condition was also significant, *F*(2, 118) = 3.80, *p* = .025, η_p_^2^ = .061. As in previous studies on the benefits of guessing, performance was higher in the immediate-feedback (*M* = .67, *SD* = .19) than in the read condition (*M* = .62, *SD* = .21), *t*(59) = 2.12, *p* = .038, *d* = 0.27. What is important, the delayed-feedback condition (*M* = .67, *SD* = .19) also outperformed the read condition, *t*(59) = 2.36, *p* = .022, *d* = 0.31.^3^ The results thus clearly show that the benefits of guessing with delayed feedback can be obtained even when study materials consist of related pairs of words.^4^ However, the interaction that was of main interest failed to emerge, *F* < 1, which indicates that the benefits of guessing with delayed feedback were equally large whether memory was ultimately tested in the novel or reinstated context.

**Table 1:** Cued-recall performance across context conditions at test and learning conditions at study in Experiments 1 and 2, averaged across blocks (standard deviations are in parentheses)

Context at Test and Guessing Condition	Reinstated			Novel			
	Read	Immediate Feedback	Delayed Feedback	Read	Immediate Feedback	Delayed Feedback (Delayed-Reinstated)	Delayed-Novel
Experiment 1	.64 (.21)	.67 (.20)	.67 (.21)	.60 (.22)	.66 (.21)	.66 (.21)	–
Experiment 2	–	–	–	.67 (.21)	.70 (.17)	.72 (.20)	.68 (.21)

In addition to accuracy, we also analyzed how study conditions influenced participants’ predictions of their future test performance—JOLs—made when full pairs were presented for study. For this analysis, the context factor was not included because at the time of making JOLs participants could not have known which context condition each of the pairs would be assigned to on the later test. The descriptive statistics for JOLs are presented in Table 2. A one-way repeated-measures ANOVA revealed that JOLs differed across the three learning conditions, *F*(2, 118) = 14.70, *p* < .001, η_p_^2^ = .199. Even though performance was lower in the read condition than in the two guessing conditions, JOLs revealed a different trend. In fact, in the read condition they were higher than in the immediate-feedback condition, *t*(59) = 4.49, *p* < .001, *d* = 0.58, replicating previous studies that have shown that the benefits of guessing with immediate feedback tend to be underappreciated by participants (Huelser & Metcalfe, 2012; Yang et al., 2017; Zawadzka & Hanczakowski, 2019), and also numerically, although not significantly higher than in the delayed-feedback condition, *t*(59) = 1.49, *p* = .142, *d* = 0.19. This divergence between predictions of future memory performance and actual performance scores once again shows that people are generally unaware of the benefits of guessing. This is particularly evident when immediate feedback is provided, as demonstrated here and in previous studies, but it seems that to some extent the same might happen when feedback is delayed.

**Table 2:** Judgments of learning (JOLs) on a 1–5 Scale across learning conditions at study in Experiments 1 and 2, averaged across blocks (standard deviations are in parentheses)

	Read	Immediate Feedback	Delayed Feedback (Delayed-Reinstated)	Delayed-Novel
Experiment 1	2.98 (0.58)	2.78 (0.62)	2.93 (0.58)	–
Experiment 2	3.31 (0.76)	3.08 (0.74)	3.18 (0.74)	3.21 (0.78)

Interestingly, we also found a difference in the magnitude of JOLs between delayed- and immediate-feedback conditions, *t*(59) = 4.25, *p* < .001, *d* = 0.55, with JOLs being higher when feedback was delayed. This pattern is intriguing inasmuch as it suggests that the decrease in JOLs found in the immediate-feedback condition compared with the read condition might not stem solely from the presence or absence of the requirement to guess. In Experiment 2, we attempted to replicate this novel finding.

Experiment 1 provided the first demonstration that the benefits of guessing with delayed feedback can emerge when related word pairs are used as study materials. It seems likely that what made the crucial difference that enabled participants to improve their test performance over that in the read condition, a pattern absent from previous studies (Grimaldi & Karpicke, 2012; Hays et al., 2013; Vaughn & Rawson, 2012), was the presence of contexts in the memory task or, more specifically, the overlap in terms of context across various stages of learning in the delayed-feedback condition, as described next.

The manipulation of context reinstatement at test clearly influenced retrieval—in line with previous studies (e.g., Smith & Manzano, 2010; Zawadzka et al., 2018)—as evidenced by generally better performance with reinstated rather than novel contexts, albeit the effect was small. However, the lack of a significant interaction between the two factors examined in the present study—context and learning condition—suggests that context reinstatement at test was not necessary for the benefits of guessing with delayed feedback to emerge. In fact, the difference in performance between the delayed-feedback and read conditions was significant even when contexts at tests were novel and so they could not have benefitted memory at this stage, *t*(59) = 2.34, *p* = .023, *d* = 0.30. This indicates that it is not the problem with lacking effective retrieval cues at the time of the test that undermines the effectiveness of guessing when feedback is delayed. This suggests that attention should be directed instead to the feedback processing stage.

Here, one needs to consider how exactly context photographs were used in Experiment 1 in the delayed-feedback condition. Remember that in this condition participants were first presented with cues only, superimposed over contexts, and later—at the feedback stage—exactly the same contexts were presented together with both cues and targets. This means that the context taken from the guessing stage was reinstated at the time of feedback provision for all pairs in the delayed-feedback condition. This is important because when contexts are reinstated during repeated study, they can trigger *reminding* of previous study episodes for their respective items (Zawadzka et al., 2018; see Hintzman, 2011, for a discussion of reminding). In Experiment 1, it thus could be that context reinstatement during feedback presentation in the delayed-feedback condition essentially reminded participants of the earlier episode of guessing and thus re-elicited the semantic activation established previously by the process of formulating guesses. This semantic activation could then facilitate encoding of feedback very much in the same way as when guessing augments memory for feedback immediately following the guessing stage.

To directly test the hypothesis according to which reminding at the time of feedback delivery is necessary to observe the benefits of guessing with delayed feedback, in Experiment 2 we dropped the context manipulation from the final test, making all test contexts novel and so incapable of improving retrieval, and focused only on the effects of context at encoding. To this end, we manipulated context specifically when feedback was delayed. We compared a condition in which the same contexts were used for guessing and delayed feedback, as in Experiment 1, with a new condition in which the guessing and feedback stages were accompanied by different contexts. If reminding at the stage of feedback delivery was indeed responsible for the benefits of guessing with delayed feedback in Experiment 1, we should replicate this effect in the former condition, which should promote reminding, but not in the latter, in which the incidence of reminding should be reduced, thus resembling standard conditions under which guessing with delayed feedback does not occur.

## Experiment 2

### Method

#### Participants

Sixty students and graduates (age range: 19–61 years, *M* = 28.1) of various universities based in Warszawa and Łódź, Poland, participated in this experiment in exchange for course credit or monetary compensation.

#### Materials and design

All verbal materials from Experiment 1 were translated into Polish, and minor changes were introduced to some of the word pairs and the instructions to accommodate language differences. Two hundred and sixty-four pictures were used as contexts, with four of them reserved for the practice task and the remaining 260 assigned to the two experimental blocks.

**Fig. 2:**
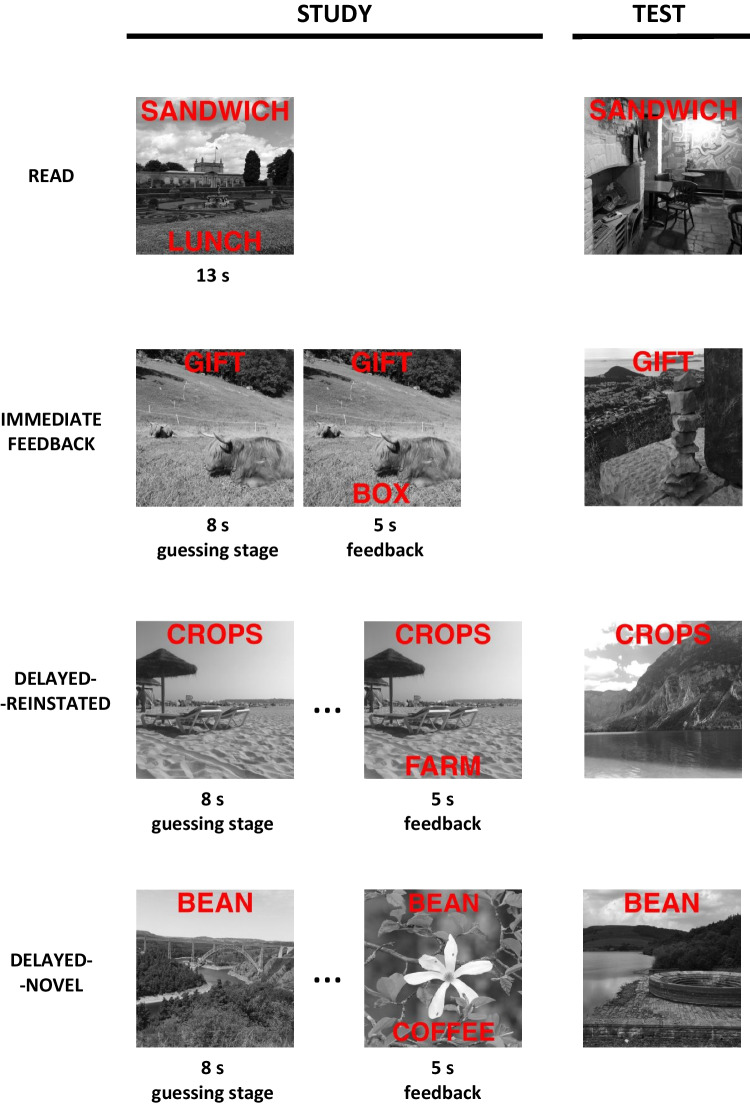
A schematic design of a single experimental block in Experiment 2. Guessing conditions are indicated on the left-hand side. Note that the actual study materials in this experiment were in Polish rather than in English

Experiment 2 excluded the context manipulation from test, as all cues at test were superimposed over novel photographs. Instead, context was manipulated when feedback was provided, but this manipulation was nested in the delayed-feedback condition that was administered to participants in two variants. The *delayed-feedback reinstated-context* (henceforth referred to *as delayed-reinstated*) condition was the same as the delayed-feedback condition in Experiment 1: participants were presented with a cue superimposed over a context photograph for guessing, and later provided with feedback superimposed over the same photograph. The *delayed-feedback novel-context* (*delayed-novel*) condition differed only in the type of photograph used for feedback: in this condition, feedback was superimposed over a novel photograph, not used before or after in the experiment. The study phases for the read and immediate-feedback conditions were the same as in Experiment 1. There were 15 pairs per block (30 in total) assigned to each of the four conditions. The average lag between the guessing and feedback delivery stages for both delayed-feedback conditions was 50 trials. The design of this experiment is presented in Fig. 2.

#### Procedure

The procedure was the same as in Experiment 1, barring some slight changes to the instructions and the practice task that were required to accommodate the addition of the delayed-novel condition.

## Results and discussion

Descriptive statistics for cued-recall performance are presented in Table 1. We entered the data from all learning conditions—read, immediate feedback, delayed-reinstated, and delayed-novel—into a one-way repeated-measures ANOVA, which revealed that learning condition did affect subsequent memory performance, *F*(3,177) = 3.41, *p* = .019, η_p_^2^ = .055. Planned comparisons revealed that, replicating the main novel result from Experiment 1, performance was better in the delayed-reinstated condition than in the read condition, *t*(59) = 3.19, *p* = .002, *d* = 0.41. Importantly, there was also a significant difference between the two delayed-feedback conditions, with performance being better when context at the stage of feedback presentation was reinstated rather than novel, *t*(59) = 3.14, *p* = .003, *d* = 0.41. In fact, performance levels did not differ significantly between the delayed-novel and read conditions, *t* < 1, thus replicating the usual lack of benefits of guessing with delayed feedback (Grimaldi & Karpicke, 2012; Hays et al., 2013; Kornell, 2014; Vaughn & Rawson, 2012) despite the presence of context pictures in the experiment. This confirms that it was context reinstatement at the stage of feedback delivery that was responsible for the benefits of guessing with delayed feedback in Experiment 1. Although not relevant for the main purpose of the present experiment, one unexpected result was the lack of a significant difference between the immediate-feedback and read conditions—the hallmark of the guessing benefits, *t*(59) = 1.65, *p* = .105, *d* = 0.21—even though the difference was in the expected direction.^5^ This result stands in contrast to the findings from previous studies on the benefits of guessing with feedback, as well as to the results of Experiment 1.^6^

Another one-way repeated-measures ANOVA was performed on JOL data, presented in Table 2, and demonstrated that JOLs were also reliably affected by the learning condition, *F*(3, 177) = 12.47, *p* < .001, η_p_^2^ = .175. As in Experiment 1, participants’ predictions starkly diverged from their actual performance. Even though performance was numerically the poorest in the read condition, JOLs in this condition were higher than in any of the remaining three conditions, *t*(59) = 3.02, *p* = .004, *d* = 0.39 against the delayed-novel condition, *t*(59) = 3.52, *p* < .001, *d* = 0.46 against the delayed-reinstated condition, and *t*(59) = 5.41, *p* < .001, *d* = 0.70 against the immediate-feedback condition. There was also no significant difference between the two delayed-feedback conditions despite a difference in memory performance, *t* < 1. This suggests that participants did not appreciate the encoding benefits afforded by reminding in the delayed-reinstated condition. Finally, JOLs were found to be lower in the immediate-feedback condition than in either of the delayed conditions, *t*(59) = 2.75, *p* = .008, *d* = 0.36, for the delayed-novel, and *t*(59) = 2.99, *p* = .004, *d* = 0.39, for the delayed-reinstated condition, replicating and extending the novel finding from Experiment 1.

The results of Experiment 2 once again show that guessing with delayed feedback can improve memory for related pairs. Our results indicate that for these benefits to emerge, the dynamics of feedback encoding are paramount. Improved memory compared with read pairs was found only when delayed feedback was presented with the context that had been earlier used with the same cue in the guessing stage—that is, when contexts were reinstated rather than novel at the stage of feedback delivery. Given that context reinstatement can trigger reminding of previous experience with the same study materials (Zawadzka et al., 2018), we argue that reinstated context at the time of feedback helps bring participants back mentally to the time of guessing. We further stipulate that reminding serves here to re-elicit the semantic activation that originally resulted from the process of guessing, which in turn facilitates either direct linking of feedback to the cue (Grimaldi & Karpicke, 2012) or creating a mediated link via previously formulated and now spontaneously retrieved guesses (Vaughn & Rawson, 2012). What our results thus clearly show is that the lack of benefits of guessing with delayed feedback found in previous studies that used related word pairs (Grimaldi & Karpicke, 2012; Hays et al., 2013; Kornell, 2014; Vaughn & Rawson, 2012) can be remedied by improving the conditions of delayed feedback processing.

## General discussion

In two experiments we have demonstrated that guessing with delayed feedback can benefit future test performance even under conditions under which previous studies (Grimaldi & Karpicke, 2012; Hays et al., 2013; Kornell, 2014; Vaughn & Rawson, 2012) have failed to show this benefit—that is, when weakly related word pairs are used as study materials. We have shown that those benefits appear if reminding of information from the guessing stage is facilitated via context reinstatement at the time of feedback delivery. This finding puts constraints on potential explanations of the benefits of guessing. In addition, it also provides novel insights into the process of reminding.

Starting with the existing accounts of guessing benefits, one needs to note that thus far these were developed to account for the lack of benefits when feedback is delayed, at least when related word pairs were used as materials. Given that this benchmark dissociation of guessing effects depending on the timing of feedback has been questioned by the present study, it is vital to examine how these accounts can be augmented to describe the benefits of guessing with delayed feedback under conditions facilitating reminding during feedback processing. We argue that some of the existing accounts struggle to account for the present findings, specifically those that invoke what can be termed motivational states as a locus of the benefits of guessing.

First, it has been proposed that what drives the benefits of guessing is curiosity, by which making a guess makes participants more eager to learn the correct answer, resulting in greater attention towards feedback and hence its better encoding (Potts et al., 2018). However, our results do not seem to sit well with the curiosity account of the benefits of guessing. The curiosity account highlights the state directly preceding feedback processing, with curiosity arising at the time of guessing and facilitating encoding of information that resolves curiosity. While it is easy to assume that curiosity is sustained for a very brief period of time before immediate feedback is provided, it is much harder to imagine that participants would be curious about arbitrary responses to word cues presented a number of learning trials earlier. The concept of curiosity puts stress on processes operating in the *anticipation* of feedback, yet the results from our Experiment 2, when the benefits of guessing were obtained with feedback presented in reinstated but not novel contexts, suggest that it is *processing *of feedback itself that matters, not the state of tension that precedes it, which was equated between the delayed-reinstated and delayed-novel conditions. Thus, while curiosity may contribute to allocating attention towards feedback, our results suggest that it is not necessary as a mechanism of the benefits of guessing.

Second, the surprise account would argue that guesses serve as explicit predictions which, when negated by the correct response, result in a state of surprise, which in turn leads to increased attention to feedback, resulting in improved memory (e.g., Potts & Shanks, 2014). Although it has been recently questioned whether guesses can be treated as predictions (Brod, 2021), the surprise account remains viable as long as it is not demonstrated that guesses are formulated with very low confidence, something the present study did not aim to do as we opted for collecting JOLs rather than confidence-in-guesses judgments. Nonetheless, the surprise account still struggles to account for the present findings because—as the curiosity account—it puts stress on the phase immediately preceding the provision of feedback. Only when the erroneous prediction directly precedes feedback can one expect surprise when corrective feedback is revealed. With delayed feedback, though, any predictions participants may formulate when guessing are decoupled from feedback processing. Like the curiosity account, the surprise account would also have trouble explaining why contexts at the time of feedback delivery can lead to the guessing benefits when they are reinstated, but fail to affect memory when they are novel.

Still, we do not wish to argue that curiosity or surprise cannot play a role in producing the benefits of guessing. Recent research suggests that these benefits are not unitary and different mechanisms may operate when there are obvious pre-experimental semantic associations between cues and to-be-learned materials and when there are none—as in the case of translation of foreign language words (Seabrooke et al., 2019)—or when these are discovered or not at the time of guessing (Zawadzka & Hanczakowski, 2019). Different loci of the effects for different types of materials may well indicate involvement of different mechanisms. If so, then it is possible to argue that while curiosity can potentially explain benefits for target memory when information lacking preexisting semantic links is to be learnt, it is either supplanted, or at the very least augmented by some other process, as a mechanism behind the guessing benefits when related word pairs are to be mastered.

Out of the potential explanations of the benefits of guessing currently under consideration, the present results are most consistent with various formulations of the semantic elaboration account, which served as the basis for the current work. First, searching one’s memory for a viable guess can activate a number of associates of the cue. When feedback is later presented, these activated associates facilitate encoding of the cue–target association, leading to a particularly rich semantic representation of this association that subsequently can be accessed by a variety of retrieval routes (Glenberg, 1979). Kornell (2014) argued that this activation of the semantic network might play more of a role for trivia than for related word pairs because the associations evoked for trivia questions are likely to be richer and more meaningful, resulting in more distinctive memory representations that can be later retrieved when delayed feedback is presented. Second, guessing by its very nature activates one associate of a cue in particular—the guess itself—and when this guess is encoded along the cue–target association, it can then serve as a mediator when a cue is presented and its target needs to be retrieved (see Carpenter, 2009, 2011).

We believe that both versions of the semantic elaboration account are consistent with our results concerning the role of reminding—which refers to spontaneous retrieval in a memory task (e.g., Hintzman, 2011)—in benefitting guessing after a delay, and that they are not mutually exclusive. Reminding could reinstate from long-term memory at least some of the activation and/or elaboration of the cue generated at the time of guessing. In this way, participants could more effectively embed the feedback within the cue’s semantic network when it is presented, which would later improve test performance. Reminding could also help participants retrieve their guesses or other potentially useful associations formulated at the time of guessing, linking them to the feedback and enabling them to be used as additional cues for target retrieval at test (see Metcalfe & Huelser, 2020, for a similar episodic recollection account of the benefits of guessing, which, however, focuses mostly on reminding at test). This could further allow for more effective semantic processing of feedback in the context of the cue when the two are finally presented together, leading to better performance at test. Note that these mechanisms—and nothing precludes their simultaneous operation at the time of feedback delivery—would make the delayed-reinstated condition very much like the standard immediate-feedback condition.

While we believe that both variants of the semantic elaboration account are equally consistent with the reminding results of our study, we also argue that the variant pointing to multiple activated associates may be more compatible with the results of Experiment 1, where the benefits of context reinstatement at test were independent of the benefits of guessing. One could argue that encoding multiple associates leads to decontextualization of knowledge (Glenberg, 1979; Smith & Handy, 2014), precisely because it allows for multiple retrieval routes. However, a more thorough examination of the issue would be required in future studies.

Independent of the particular mechanism responsible for the benefits of guessing, it is apparent that these benefits when feedback is delayed are achieved with the help of another process: reminding. Reminding has been postulated as a mechanism behind the benefits of spaced learning (e.g., Benjamin & Tullis, 2010), and is also known to influence performance in the guessing paradigm (Metcalfe & Huelser, 2020). Importantly, reminding is known to aid memory when participants are reminded of being previously presented with the same (Zawadzka et al., 2018) or related (Tullis et al., 2014) study items. This is generally linked to a wide class of phenomena related to memory retrieval generally—retrieving information from memory strengthens its representation and thus also facilitates future memory performance (e.g., Karpicke & Roediger III, 2007; Kornell et al., 2011).

However, the benefits of reminding in the present study come with an important twist. In our study, reminding improved memory even though it entailed retrieval of related but *different* information, when matching contexts triggered retrieval of the guessing episode during which the correct answer was not even presented. Such observations of memory improvement due to retrieval of related information are scarce in the literature. Tullis et al. (2014) found such benefits of reminding, but only for information participants were reminded of and not for information that triggered reminding. Wahlheim and colleagues (Wahlheim et al., 2014; Wahlheim & Jacoby, 2013) reported memory benefits for information for which participants explicitly reported being reminded of related information. This effect, however, was countered by interference from this related information when participants did not report reminding. Our results, on the other hand, show general benefits of reminding for encoding of related information even when results are not conditionalized on explicit reports of reminding. By this, they unequivocally demonstrate that reminding creates fertile ground for additional encoding of novel information that becomes embedded in the knowledge base activated by spontaneous retrieval, creating an integrated memory representation. This case resembles forward effects of explicit testing, by which being tested on some part of materials facilitates encoding of additional information (see Chan et al., 2018, for a review), documenting once again the importance of retrieval for new learning.

Our final considerations concern participants’ insights into the benefits of guessing. Whenever conditions are found that facilitate learning, it is worth asking whether people are generally aware of the memory benefits they convey, because such awareness is likely to determine their willingness to adopt effective learning strategies. To this end, we also included in the present study a measure of how participants perceive the effectiveness of their own learning. Previous research that looked at the same measure in the context of guessing with immediate feedback found that participants underestimate the benefits yielded by initial guessing (Huelser & Metcalfe, 2012; Potts et al., 2018; Zawadzka & Hanczakowski, 2019). In particular, participants commonly predict better test performance when they read the to-be-remembered material outright rather than when they attempt guessing at a correct answer first. Here we looked at whether the same pattern would emerge in the case of delayed feedback or whether delaying feedback has the power of mending this particular metacognitive illusion. We fully replicated previous observations according to which participants consistently predict performance to be the best in the read condition, extending these findings to the case of delayed feedback. Given that our results also revealed memory benefits of guessing with delayed feedback, at least when context was reinstated at the time of feedback presentation, our results suggest that participants’ belief that guessing harms one’s memory constitutes a metacognitive illusion that is particularly pervasive.

However, as also pointed out by the present set of results, the degree of this illusory belief in the harmful effects of guessing on memory can vary. Our novel finding was that participants provided lower JOLs when feedback was presented immediately after the guessing attempt than after a delay. Importantly, this pattern was found despite comparable memory performance between the immediate and delayed feedback conditions across the two experiments reported here, and in Experiment 2 was unaffected by whether delayed feedback was presented with reinstated or novel contexts despite a difference in memory performance between these conditions. What we believe these results suggest is that the commonly found decrease in JOLs in the immediate-feedback compared with the read condition has two distinct causes. First, it is a reflection of participants’ belief that learning by generating errors is an inferior strategy compared with reading (Yang et al., 2017). As our results show, this belief can be easily extended to the delayed-feedback conditions. Second, and perhaps more interestingly, there is an experiential component to this illusion as well. Most likely this is due to the fact that being presented with negative feedback right after an incorrect guess is a particularly aversive experience, as it underscores straight away the gap between one’s own answer and the correct one. It could be assumed here that when feedback is delayed, participants’ commitment to their earlier guesses is somewhat lessened, and likely not all guesses are even remembered at this stage (see Butowska et al., 2022, for an interim test of memory for guesses). Thus, our results are consistent with a dual-basis account of JOLs (Koriat et al., 2004).

Interestingly, it has to be noted both of the observations discussed above hold true even when the fact that our procedure was done in blocks is taken into consideration. As mentioned before, study–test block did not interact with any other condition across our analyses, and for JOLs there was also no main effect of block in either of the experiments. This means that JOLs were misaligned with memory performance despite participants having a chance to experience the benefits of guessing first-hand in the first test. Given that inaccurate metacognitive monitoring of learning can hinder the use of effective learning techniques, possible interventions that could help mend this particular illusion are a vital avenue for future studies.

## Figures and Tables

**Fig. 3:**
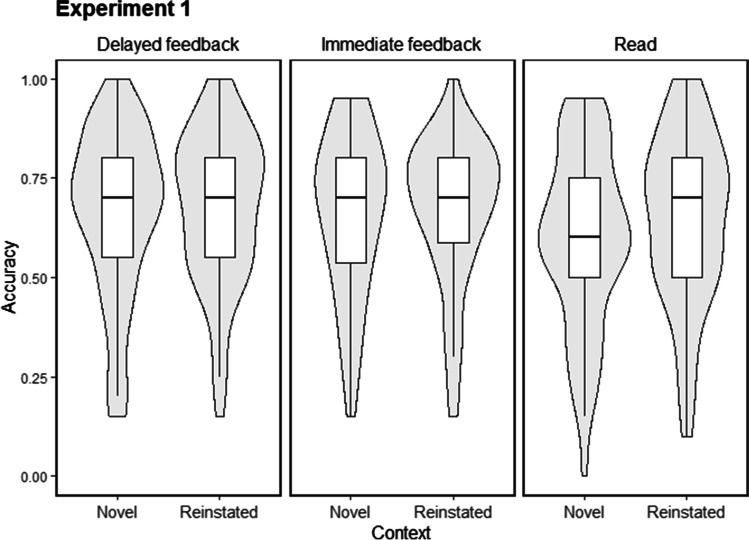
Cued recall performance across context conditions at test and guessing conditions at study in Experiment 1. Boxplots represent group-level data and violin plots depict density of individual participants’ accuracy scores

**Fig. 4:**
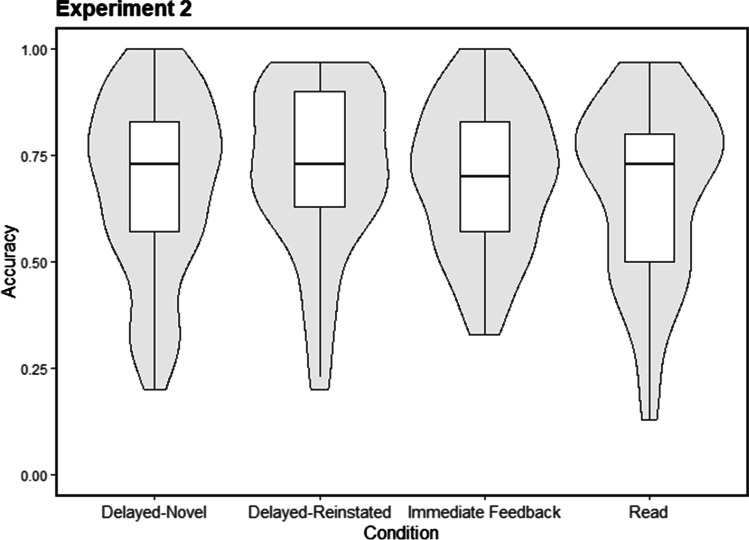
Cued recall performance across conditions in Experiment 2. Boxplots represent group-level data and violin plots depict density of individual participants’ accuracy scores

## Appendix

Box and violin plots for cued recall performance in Experiments 1 and 2

### Author note

Katarzyna Zawadzka, Faculty of Psychology and Cognitive Science, Adam Mickiewicz University, and Interdisciplinary Center for Applied Cognitive Studies, SWPS University; Oliwia Zaborowska, Ewa Butowska, and Krzysztof Piątkowski, Interdisciplinary Center for Applied Cognitive Studies, SWPS University; Maciej Hanczakowski, Faculty of Psychology and Cognitive Science, Adam Mickiewicz University.

This work was supported by the National Science Centre (Poland) grants 2017/27/B/HS6/02001 awarded to Maciej Hanczakowski, and 2018/29/B/HS6/01313 awarded to Katarzyna Zawadzka, and by grant PPN/PPO/2018/1/00103 from the National Agency for Academic Exchange (Poland) awarded to Katarzyna Zawadzka.

## Footnotes

1 The results of Zawadzka and Hanczakowski (2019) suggest that eliciting versus not eliciting JOLs in the paired-associates version of the guessing task should have no impact on test performance—that is, that no JOL reactivity should be expected.

2 These trials varied in duration and their number typically included a combination of read trials, guessing-with-feedback trials from the immediate-feedback condition, and guessing trials as well as feedback trials from the delayed-feedback condition.

3 In studies on guessing, two types of analyses are typically reported: with and without items that were guessed correctly. In our study, we were interested more in the consequences of guessing with delayed feedback than in the consequences of *incorrect* guessing. After all, when guessing is used as an educational strategy, some guesses are bound to be correct. Nevertheless, because some readers might be more interested in the effects of incorrect guessing, we also present analyses after the exclusion of trials on which participants’ guesses were correct, which constituted 3.5% of all trials for which participants had to provide a guess. The same ANOVA on incorrectly guessed items only revealed a similar pattern as the main analysis. The main effect of context was significant, *F*(1, 59) = 5.53, *p* = .022, η_p_^2^ = .086, with reinstated context (*M* = .66, *SE* = .02) leading to better performance than novel context (*M* = .64, *SE* = .02). The main effect of learning condition was also significant, *F*(2, 118) = 3.14, *p* = .047, η_p_^2^ = .051, but the interaction was not, *F*(2, 118) = 1.02, *p* = .365, η_p_^2^ = .017. Performance was better in the delayed-feedback condition (*M* = .66, *SD* = .19) than in the read condition (*M* = .62, *SD* = .21), *t*(59) = 2.23, *p* = .030, *d* = .29, and it was also marginally higher in the immediate-feedback condition (*M* = .66, *SD* = .19) than in the read condition, *t*(59) = 1.77, *p* = .082, *d* = 0.23.

4 Given that the procedure consisted of two separate study-test blocks for different sets of materials, we also conducted ANOVAs with block as an additional factor. As there were no interactions between block and any of the other factors in either of the experiments, we do not report these analyses.

5 In this experiment, correct guesses were provided on 2.8% of trials from the three conditions that required participants to guess the target. An ANOVA performed on data after the exclusion of those trials on which participants guessed the targets correctly was marginally significant, *F*(3, 177) = 2.39, *p* = .070, η_p_^2^ = .039. Planned comparisons revealed that even in this restricted dataset performance in the delayed-reinstated condition (*M* = .71, *SD* = .20) was better than in the read condition (*M* = .67, *SD* = .21), *t*(59) = 2.54, *p* = .014, *d* = 0.33, and the delayed-novel condition (*M* = .67, *SD* = .21), *t*(59) = 2.81, *p* = .007, *d* = 0.36. There were no significant differences between the read condition and the immediate-feedback condition (*M* = .69, *SD* = .18), *t*(59) = 1.21, *p* = .230, *d* = 0.16, nor between the read and delayed-novel conditions, *t* < 1.

6 Although we initially collapsed the data across blocks for all analyses, the surprising lack of the usual benefits of guessing with immediate testing made us look closer at performance across blocks. The guessing benefit was present when only data from the first block were analyzed, *t*(59) = 2.17, *p* = .034, *d* = 0.28, with higher performance for the immediate feedback (*M* = .68, *SD* = .19) than in the read condition (*M* = .63, *SD* = .24).This effect was, however, absent from the results of the second block, *t*(59) = 0.43, *p* = .667, *d* = 0.06, with comparable performance in the immediate feedback (*M* = .72, *SD* = .21) and the read condition (*M* = .70, *SD* = .22). This pattern is reminiscent of the results obtained in studies examining the generation effect, where it has been argued that participants adopt more effective learning strategies in the control read condition on the second block (deWinstanley & Bjork, 2004). It is thus possible that the blocked design undermined our ability to provide a clear demonstration of the benefits of guessing with immediate feedback. For now, this is only a tentative suggestion inasmuch as there was no similar pattern of differences across blocks in Experiment 1 and the interaction of learning condition and block was not reliable in Experiment 2, and so the issue requires future research. We thank Jennifer S. Burt for suggesting these analyses.
